# Ovarian Tumour Cured by Puncture and Irritating Injections

**Published:** 1831

**Authors:** 


### XXXI.

### Ovarian Tumour cured by Puncture and Irritating Injections.

A case of this kind is detailed in the Transactions of the Medical Society of Lyon, by M. Rigollot. The patient was 23 years of age, who experienced, after an accouchement, an attack of uterine inflammation, that was not very skilfully treated. The disease became chronic, attended with dull settled pain in the ovarian region, and general ailing. The abdomen became swollen, and slow consumptive fever supervened. Exciting medicines administered (under the supposition *that the disease was tympanitis*) aggravated materially all its symptoms. When M. Rigollot was called to examine the patient, her emaciation was extreme, her fever continued, the abdomen prominent in its anterior and left lateral part, and obscure fluctuation was perceptible. The tumour was punctured, and ten or twelve pounds of purulent, fœtid, and greenish fluid, escaped through the canula.

Twenty days after, a second puncture was made, which gave issue to some matter, and the cavity was then injected with a decoction of plantain and red rose leaves, with a little wine. Acute pain was endured while the injection remained in the cyst. After its entire discharge, the surgeon kneaded the cyst with his fingers, in order to determine its inflammation. Intense pain, vomiting, and swelling of the abdomen quickly ensued, and were energetically treated with antiphlogistic measures. In a month the cure was complete. The patient, nevertheless, retained in the abdomen a small, oblong indolent tumour, doubtless formed by the adherent parietes of the cyst.

We would caution junior and "bold surgeons" against too confident expectations from tapping and injecting ovarian cysts. We have witnessed some of these operations; but the results were not of an encouraging nature. When we reflect on the well known fact, that these cysts are almost always divided into compartments, having little or no communication with each other, it will be evident that we cannot evacuate them by puncture; and that to excite inflammation in one or two of these compartments will only tend to increase effusion into the others. Besides, the inflammation itself of the cyst is no trifling danger. In most of the operations which we have witnessed, the patients died of this inflammation—even from common puncture. Within these few days (5th August) we witnessed a fatal case of inflammation of the cyst of an ovarian tumour without any operation; and as the case is curious we shall here state a few of the particulars. The patient was a lady about 35 or 38 years of age, who had a tumour in the centre of the abdomen, which was perfectly circumscribed, excepting inferiorly. The early history of the case could not be obtained, and therefore it was not ascertained whether or not the tumour originated in one side of the abdomen. Two or three times this tumour appeared to be the seat of acute inflammation, and required very active depletion. A similar attack occurred early in August last, and Mr. Skair, of Castle-street, employed the same means as formerly used, viz. copious depletion, but without the same success. Instead of a speedy cessation of pain, as formerly the result of venesection, the patient fell into a state of exhaustion, with laborious breathing, cold extremities, and great restlessness. From this state she could not be roused by cordials, and she died in less than 24 hours. Mr. Thomas, Mr. Skair, and Mr. Nicholson, examined the body, and we were present at the examination. The tumour resembled a uterus in the sixth or seventh month of pregnancy. It was almost black, so completely injected were the vessels of the cyst. In short, it looked as if it were in a state of incipient gangrene. Yet, strange to say, there was not the slightest adhesion between it and the surrounding intestines, nor any effusion of fluid in the abdomen. No other part was inflamed except the sac of the ovarian tumour, which rose by a very narrow peduncle from one of the fallopian tubes. The other ovary was a solid scirrhus, the size of a small apple, and the uterus itself was *scirrhous.* The cyst contained several compartments, most of which were filled with a fluid resembling blood and water. The others contained solid and semi-solid contents of various kinds.

This case shews that an inflamed cyst may occasion death, without the inflammation spreading to any other organ or part. Therefore the exciting of inflammation in an ovarian cyst is a matter of no trifling danger. The circumstance of the ovarian tumour having gained such a size without any adhesions, and also the fact of its being connected, up to the period of death, by only a small peduncle with the uterus, are favourable to the proposals of those who have attempted and recommended extirpation of diseased ovaries. No case could be more favourable for such an operation than this, as far as the *execution* of the operation is concerned:—and yet it would have been unsuccessful in the end, on account of the scirrhous disease of the other ovarium and of the uterus — diseases which would, no doubt, have been called into activity by such a formidable operation as gastrotomy.

---

XXXII.

